# Metabolic Support in Acute Respiratory Distress Syndrome: A Narrative Review

**DOI:** 10.3390/jcm12093216

**Published:** 2023-04-29

**Authors:** Michele Umbrello, John J. Marini, Paolo Formenti

**Affiliations:** 1 Unità Operativa di Anestesia e Rianimazione II, Ospedaliera San Carlo, ASST Santi Paolo e Carlo, 20148 Milan, Italy; 2 Department of Pulmonary and Critical Care Medicine, University of Minnesota, Minneapolis, MN 55455, USA; 3 SC Anestesia, Rianimazione e Terapia Intensiva, ASST Nord Milano, Ospedale Bassini, 20097 Cinisello Balsamo, Italy

**Keywords:** metabolic support, acute respiratory distress syndrome, artificial nutrition

## Abstract

Nutritional support for acute respiratory distress syndrome (ARDS) patients shares metabolic notions common to other critically ill conditions. Nevertheless, it generates specific concern regarding the primary limitation of oxygen supply and the complications of carbon dioxide elimination, as well as the significant metabolic alterations due to the body’s response to illness. In the present narrative review, after briefly summarizing the pathophysiology of critical illness stress response and patients’ metabolic requirements, we focus on describing the characteristics of metabolic and artificial nutrition in patients with acute respiratory failure. In patients with ARDS, several aspects of metabolism assume special importance. The physiological effects of substrate metabolism are described for this setting, particularly regarding energy consumption, diet-induced thermogenesis, and the price of their clearance, transformation, and storage. Moreover, we review the possible direct effects of macronutrients on lung tissue viability during ARDS. Finally, we summarize the noteworthy characteristics of metabolic control in critically ill patients with ARDS and offer a suggestion as to the ideal methods of metabolic support for this problem.

## 1. Introduction

Nutritional support for patients with acute respiratory distress syndrome (ARDS) has metabolic notions in common with other categories of critically ill patients. Nevertheless, it may generate additional concern due to the limitations of tissue gas exchange associated with the syndrome. Different metabolic phases follow each other during an ICU stay [1,2]. The early period is characterized by metabolic instability and a severe increase in catabolism. The later period is characterized by significant muscle wasting and stabilization of the metabolic disturbances. Finally, the post-acute phase follows with improvement and rehabilitation or persistent inflammatory catabolism. An increased secretion of pro-inflammatory cytokines [3], catabolic hormones [4], and insulin resistance [5] characterize the metabolic response to critical illness. All these cause increased glycogenolysis, gluconeogenesis and lipolysis, and augmented muscle protein breakdown, with the aim of ensuring sufficient energy and amino acids for wound repair and immune function. The main nutritional consequences of the acute stress response, which must be taken into careful consideration, are dysregulated endogenous glucose production and augmented resistance to anabolic stimuli. Ideally, the resolution of the triggering event (such as infection control or wound treatment) could reverse the stress response. Unfortunately, nutrition per se could not reverse this phase. There are limited high-quality data to determine the impact of energy overfeeding of critically ill patients. However, based on the available evidence, overfeeding does not appear to affect mortality or other important clinical outcomes. Large RCTs have shown no beneficial impact on muscle wasting, and several RCTs have shown increased ureagenesis by enhanced feeding (feeding-resistant catabolism) [6,7,8,9,10]. The appropriate caloric target for critically ill adults is unclear. For instance, in a study, the delivery of a moderate number of non-protein calories compared with the planned delivery of a full dosage of non-protein calories was not associated with a lower mortality risk [11], even among patients at high and low nutritional risk [9]. Two randomized controlled trials involving patients with acute lung injury evaluated minimal or trophic enteral feeding (15% to 25% of estimated caloric requirements) with no protein supplementation, and the reported outcomes were like those of standard enteral feeding [8,12]. One study even found that enteral provision of more than 2/3 of estimated energy and protein needs given from admission to hospital discharge increases mortality [13]. Until now, no large study has identified a subgroup benefit from early full nutritional support, nor an advantage from increased amino acid doses or from indirect calorimetry-based energy dosing targeted at full energy expenditure [14]. Moreover, both enteral and parenteral routes can safely be used to administer substrates [15], while enteral nutrition is currently preferred because it is believed to maintain the absorbent function and integrity of the intestinal barrier. However, the Nutrirea-2 study showed that early isocaloric enteral and parenteral nutrition did not differ in terms of mortality, while gastrointestinal complications were more associated with enteral nutrition [16]. On the contrary, late parenteral nutrition showed fewer infections, enhanced recovery, and lower health care costs [6]. The primary source of easily available energy for all tissues is the glucose metabolized from carbohydrates (CHO). However, stress hyperglycemia may have deleterious effects on outcomes [17], accounting for the fact that glycemic control persists as a significant target in critically ill patients. In patients with acute lung injury, the application of initial trophic enteral feeding for the first week of their ICU stay was associated with less gastrointestinal intolerance but did not show as improving ventilator-free days, mortality, or infectious complications, as compared with full enteral feeding [8]. Finally, nutrition during the recovery phase of critical illness still often remains underrated. A few studies have demonstrated suboptimal nutritional intake in ICU survivors and have identified a multitude of factors influencing nutritional recovery [18,19]. Trace elements and vitamins, named together as micronutrients (MNs), are essential for human metabolism. Recent research has shown the importance of MNs in common pathologies, with significant deficiencies impacting clinical outcomes. In fact, a depletion of vitamins or trace elements may be experienced during artificial nutrition [20]. Even if an adequate enteral intake of calories ensures the needs of microelements and vitamins, the trace elements and vitamins must be supplemented in cases of inadequate intake or parenteral support [21]. The current narrative review summarizes the available literature on the metabolic alterations and the nutritional characteristics of critically ill patients with ARDS, with the aim of providing suggestions for the metabolic treatment of these patients.

## 2. Artificial Nutrition Pitfalls in Critical Illness Patients

### 2.1. The Determinants of Energy Consumption 

Comprehensive reviews of the present topic are present in the literature [22,23,24]. The main concepts are summarized in Table 1. Briefly, different amounts of oxygen are required for complete oxidation of one mole of CHO, lipids, or proteins. Thus, 200, 212, and 239 mL of oxygen are needed to obtain 1 kcal by selectively oxidizing CHO, lipids, or proteins, respectively. Therefore, the oxygen supply needed for cardiac work is greatest when consuming lipids and least when burning CHO. On the contrary, 200 mL of carbon dioxide is produced from 1 kcal of CHO, while this is less for lipids (157 mL) and proteins (191 mL). This implies that the respiratory work is the least for lipids. Furthermore, 3.7 L of oxygen is needed to produce each mole of ATP for CHO, 3.9 L of oxygen for lipids, and 5 L of oxygen for protein. In other words, the ATP generated per liter of oxygen is maximized when oxidizing CHO. Hence, the available oxygen should be more efficiently used by burning CHO, while the reduction in carbon dioxide production comes from lipid oxidation. Nevertheless, a distinction exists regarding each gram of substratum oxidized and the energy released. Mixed CHO produces 4.18 kcal/g, mixed lipids produces 9.44 kcal/g, while protein oxidation produces 4.7 kcal/g [25].

### 2.2. Diet-Induced Thermogenesis (DIT) 

DIT means the energy required for absorbing, processing, and storing nutrients. It requires an energy expenditure (EE) increase with respect to the post-absorptive state [26]. Jequier et al. [27] examined the thermic effect of nutrients, showing how intravenous glucose and lipid infusions required an EE increase of 7% and of 3% of that calculated from the energy infused, respectively. On the contrary, the stimulation of EE was 1/4 of the energy infused as amino acids in depleted patients.

### 2.3. Resting Energy Expenditure (REE)

The estimation of EE by using predictive equations fails to match measured expenditure in up to 2/3 of patients, often overestimating the actual needs [28]. Achieving appropriate nutrition is relevant. The studies summarized in Table 2 report a possible favorable outcome associated with the amount of calories prescribed, as well as between protein intake and survival [29,30,31]. Measurement of energy expenditure is possible by applying indirect calorimetry, a technique based on the calculation of nitrogen excretion, VO_2_, and VCO2 from substrates involved in oxidative processes [32].The simplified Weir equation allows the calculation of the energy production (i.e., REE) [33]:

REE (kcal) = 3.9 × l O_2_ used (L/min) + 1.1 × l CO_2_ produced (L/min)

The main technical issue that limits its use is the lack of precision in the measurement at inspired oxygen concentrations above 60%. Another limitation is the unsteady state caused by different stores and transit times of O_2_ and CO_2_ [32,43,44]. Additional restraints occur when gluconeogenesis, lipogenesis, or ketogenesis are elevated [45]. Even with these limits, indirect calorimetry remains the best available method for the estimation of EE [46]. However, the feasibility of implementing it on a large scale is questionable. The TICACOS-International RCT was stopped prematurely because of slow recruitment, perhaps reflecting the difficulty of applying indirect calorimetry on a routine basis [47]. Very recently, a retrospective study compared the use of REE calculated by indirect calorimetry as compared with predictive formulae, both in healthy and critically ill patients [48]. The authors showed computations significantly higher in the critically ill patients with lower accuracy for the predictive formula. In the absence of an indirect calorimetry device, two different strategies to measure energy expenditure could be applied. If cardiac output is monitored with a pulmonary artery catheter, and assuming a median non-protein respiratory quotient of 0.94, Fick-derived VO_2_ × 7 yields the amount of kcal/24 h [49]. If volumetric capnography, and thus carbon dioxide production, is available, REE ≅ VCO_2_ × 8.19 [50]. In summary, the estimation of energy expenditure with complex formulae is far from being fair and accurate, whatever the complexity of the equations utilized in that effort. The adoption of the ESPEN guideline recommendation of 20–25 kcal/kg/day [51] seems a reasonable suggestion, as despite its inherent imprecision, it appears to function as well as or better than more complex equations with respect to patient outcomes [15,52]. 

### 2.4. Polyunsaturated Fatty Acids 

**Figure 1:**
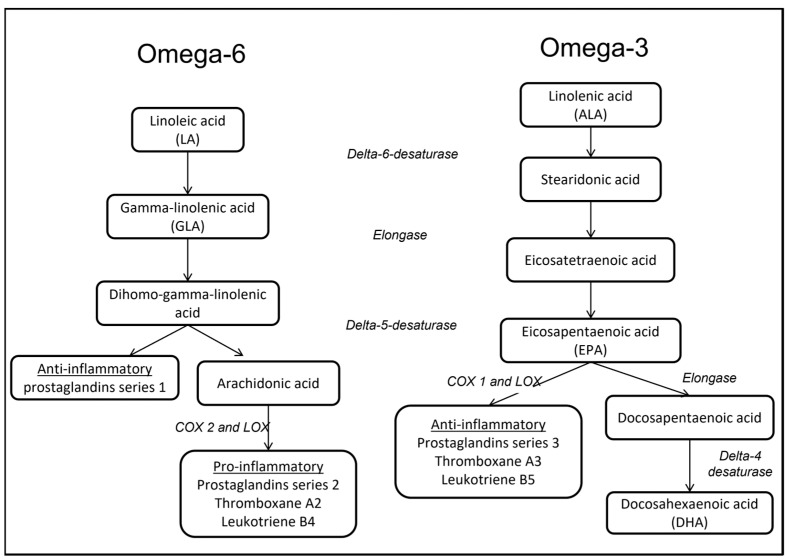
Metabolic pathways of Ω3 and Ω6 fatty acids. COX: cyclooxygenase; LOX: lipoxygenase.

Based on the location of the first double bond, polyunsaturated fatty acids can be categorized into Ω3, Ω6, or Ω9, each one with a specific biological action (Figure 1). Long-chain Ω6 fatty acids (linoleic and gamma-linolenic acid) were correlated with the pro-inflammatory phenotypes that are particularly worrisome in critically ill patients [53]. Their source may also result in increased synthesis of vasodilating prostaglandins [54]. Furthermore, they can affect lung mechanics and ventilation/perfusion regulation, worsening gas exchange [55,56]. Ω3 fatty acids—such as eicosapentaenoic and docosahexaenoic acid—could moderate inflammatory processes [57]. Their use seems to be able to shift the production of cytokines in favor of leukotrienes and trienoic prostaglandins [58]. Currently, we are unaware of any potential negative effect of the Ω9 polyunsaturated fatty acids. Protein administration leads to increased minute ventilation, suggesting an augmentation of ventilatory drive [59]. This must be considered when the work of breathing cannot be improved. Finally, in severe sepsis, attention has been drawn to the risks of enhancing the supply of arginine due to its pro-inflammatory characteristics [60].

## 3. Peculiarity of Artificial Nutrition in Acute Respiratory Failure 

ARDS is characterized by different degrees of hypoxemia and increased pulmonary permeability without cardiogenic pulmonary edema [61]. ARDS patients often present with altered respiratory system properties, with an increased and ventilatory dead space shunt fraction, impairing hypoxemia and hypercapnia [62]. From a metabolic perspective, ARDS is characterized by a pro-inflammatory response associated with hyper-catabolism [63]. Related nutritional deficits can alter lung defense mechanisms in association with respiratory muscle function [64]. In these critically ill patients, impaired lung and often cardiac function lead to elevated resting energy expenditure (REE) over 100% of that predicted by their body mass, increasing oxygen consumption (VO_2_) and carbon dioxide production (VCO_2_) [65,66]. Parenteral nutritional support with high levels of CHO increases body temperature respiratory quotient, VCO_2_ [67], and ventilatory demand, as suggested by a small uncontrolled study [68] and a small RCT [69]. However, enteral CHO has been associated with improved clinical outcomes [70] and better muscle protein accumulation [71]. Due to a scarce utilization of fat due to impaired oxidation and inefficient transport between nutrient pools, carbohydrates appear to be the preferential substrate in critical illness [72]. Indeed, the potential development of endotoxemia may develop in high-fat diets due to changes in gastrointestinal barrier function or microbiota composition [73]. Therefore, since ARDS patients either experience or are predisposed to infections, the use of a high-fat diet is contraindicated. Interestingly, Ω3 fatty acid supplementation has been experimentally associated with restored permeability of an injured alveolar–capillary membrane and with lower levels of tissue inflammation [74].

## 4. Metabolic Control in ARDS Patients

**Figure 2:**
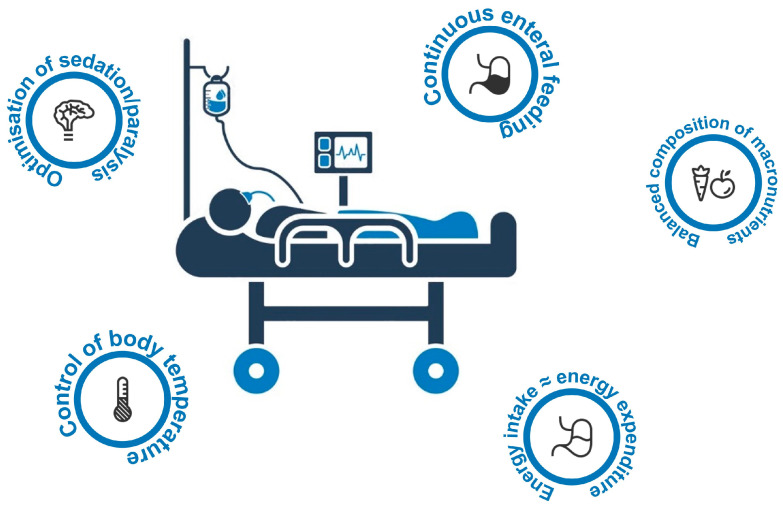
Suggestion for optimal metabolic support of patients with Acute Respiratory Distress Syndrome (ARDS).

The available guidelines suggest administration of nutritional support to ARDS patients who undergo mechanical ventilation [51]. Observational studies [37] and RCTs [47,75,76] in mechanically ventilated patients have reported the clinical benefits of prescribing an energy supply based on indirect calorimetry. However, metabolic support for this category of patients remains tricky. Figure 2 summarizes a few suggestions. Firstly, a reduction in metabolic demands can be achieved in different ways, such as reducing physical activity, controlling body temperature, and avoiding the provision of energy intake greater than needed [77,78,79]. Interestingly, the increase in caloric provision has been correlated with VCO2 [80]. The suggested energy supply is 25 kcal/kg/day; however, given the presence of a significant (although difficult to measure) amount of endogenous glucose production, this target should be reached gradually over the first week of an ICU stay. Indeed, energy capacity can be reduced to even less than 15 kcal/kg when gas exchange is severely impaired; then, the use of continuous feeding may confer an advantage from a metabolic point of view as DIT is minimal during enteral feeding [81,82]. However, when the energy supply approaches twice the REE, DIT increases up to 20% of the total EE. These observations have been attributed to the fact that digestion and absorption of nutrients cost energetically less than nutrient storage [83]. Oral administration of nutrients causes a higher thermogenic effect than a continuous enteral supply, while VO2 and VCO2 are essentially reduced even with continuous PN [84]; then, the components of macronutrients may be modified. Doing so can help minimize the need for mechanical ventilation, possibly manipulating VO_2_/VCO_2_ and DIT. Nonetheless, macronutrient components are estimated to have much less of an impact on carbon dioxide production when the design of the nutrition support program approaches the energy requirements [85]. Thus, we should abandon the traditional suggestion that more than half of the non-protein portion of enteral caloric intake should consist of lipids to reduce VCO2 and minute ventilation. Parenteral administration of Ω6 linoleic acid seems to be detrimental in patients with severe pulmonary failure [86,87]. Several studies have reported how the use of Ω3 fatty acids may confer biochemical and clinical advantages by modifying the metabolic stress response and modulating immunity and inflammation [55,88,89]. Other authors [36,90] and a more recent meta-analysis [91] have shown no significant reduction in ARDS mortality nor in ventilator- and ICU-free days when an immunomodulatory diet was supplied. However, these studies have significant heterogeneity and study design biases such as giving a relatively high amount of Ω6 fatty acids in the control group. Consequently, the guidelines on nutrition support in ARDS patients recommended providing an enteral/parenteral formula with balanced Ω6, Ω3, and Ω9 long-chain fatty acids and avoiding enteral formula with an anti-inflammatory lipid profile and antioxidants [61,92,93]. The role of glutamine supplementation is highly controversial, even if the ASPEN/SCCM guidelines no longer suggest glutamine by the enteral or parenteral route [85]. This was suggested by recent negative results seen with the MetaPlus trial [94] and the REDOX Trial [95], as well as several RCTs that showed no benefit [96,97,98]. In summary, limited administration of substrates in the first days of the early phase of illness is acceptable and possibly necessary if both endogenous energy production and resistance to anabolic signals are considered. Nutritional support then must be gradually increased to reach the targets in the following days.

## 5. Special Issues Related to SARS-CoV-2 Infection

In COVID-19 patients, the impact of inflammation and the often extended length of hospital stay may aggravate the baseline nutritional level, leading to increased disability, mortality risk, and reduced quality of life [99,100]. The risk of malnutrition is especially high in elderly patients, with multiple comorbidities, and those with a loss of skeletal muscle and reduced mobility [101,102]. Regarding critically ill COVID-19 patients, artificial ventilator assistance may average two weeks [98], thereby increasing the already high nutritional risk [103,104]. In COVID-19-related ARDS, adequate nutritional support can reduce the inflammatory pattern by helping the immune system and avoiding malnutrition, possibly facilitating ventilator weaning [105]. On the other side, critically ill COVID-19 patients are at a higher risk of sepsis and multiple-organ failure. Gastrointestinal functions are affected by high-PEEP ventilation, certain medications, and immobility [106]. For these reasons, malnutrition screening is needed in all COVID-19 ARDS patients, with a NUTRIC score greater than 5 (without IL-6 dosage) having been proposed as a threshold for a high nutritional risk [107]. Nasogastric tube feeding is considered the standard approach for artificial nutrition, and the latter should be delivered with an infusion pump, with a slow start and an incremental infusion rate based on individual tolerance [108]. Supportive or total PN, along with daily micronutrient and vitamin supplementation, should be considered if enteral nutrition is not sufficient. Central venous access is needed if PN is needed for >15 days, while peripheral access is sufficient if the duration of PN is <15 days or is provided in the lower dosages supportive of enteral nutrition [109]. During the non-invasive ventilation support of patients who are not able to eat, enteral nutrition is generally delivered by a nasogastric feeding tube, given the inability to eat by mouth. Enteral feeding in this circumstance is often difficult or risky; however, the gastric tube itself may be associated with air leakage that compromises the effectiveness of non-invasive ventilation, or it can lead to gastric distension, affecting diaphragmatic function and posing a hazard of regurgitation [110]. In these patients, given the long course of ventilator support, supportive PN has been proposed as a strategy to limit the interruptions of assisted ventilation during meals [111]. Regarding oral nutrition, frequent and small meals are suggested, and they should be supplemented with calorie- and protein-dense nutritional supplements in powder or liquid form. COVID-19 patients often experience loss of appetite, dysphagia, and gastrointestinal symptoms [112]. Gastric residual volumes greater than 500 mL/6 h and a higher risk of aspiration and nausea are factors that may suggest the positioning of post-pyloric nasojejunal tubes for 6 h [113]. Like other non-COVID-19 ARDS patients, energy expenditure could be determined by indirect calorimetry, with the already-mentioned limitations. In case of unavailability, it can be predicted as VCO_2_ × 8.19, using carbon dioxide production derived from the ventilator, or as VO_2_ × 7, using oxygen consumption estimated using data from a pulmonary arterial catheter [114]. In all the other situations, a simple, weight-based predictive formula of 20–25 kcal/kg/day is used, using the actual body weight if the BMI <30 Kg/m^2^, or the adjusted body weight (i.e., the ideal body weight + (actual body weight – ideal body weight) × 0.33) if the BMI exceeds 30 Kg/m^2^ [115]. Marginally hypocaloric nutrition (about 70% of the estimated needs) should be provided during the first week, before achieving the targeted 80–100%, due to the higher risk of overfeeding associated with the use of predictive equations [105]. Protein requirements are about 1.3 g/Kg/day. However, as with energy provision, that goal should be achieved gradually within 3–5 days [108]. Glucose/carbohydrates should not be given at rates greater than 5 mg/Kg/min or 3–4 mg/Kg/min in case of pre-existing hyperglycemia. Intravenous lipid infusions should not exceed 1.5 g/Kg/day [116]. Nutritional formulae enriched with Ω3 can be considered [117], and severely ill patients with low vitamin D blood levels (<12.5 ng/mL) can be supplemented [118]. Extracorporeal supply or prone position should not limit or contraindicate EN [119]. Finally, after resolution of a critical illness, patients have been shown to experience high rates of dysphagia that can persist after critical phases, limiting nutritional intake [120]. Consequently, nutritional modifications are often necessary to adjust the food consistency to the swallowing capability, and nutritional counseling is often required after ICU discharge.

## 6. Conclusions

In summary, nutritional support for patients with ARDS may be provided from an early stage of hospitalization when patients are hemodynamically stable (even if still under vasoactive drugs) and blood gases are adequate. The prescription for nutritional support should consider the underlying metabolic modifications of patients with ARDS, such as endogenous glucose production and anabolic resistance. It should then start at a low rate and be increased gradually. Energy may be given in amounts equal to REE (possibly measured by indirect calorimetry) or not greater than 25 kcal/kg, provided by a balanced CHO/lipids formula, possibly coupled with insulin. Moreover, an adequate amount of protein should be provided, even if its cost in terms of VO_2_/VCO_2_ is significant. Indeed, this approach may not be able to fully reduce the utilization of endogenous stores and does not possess the same protein-sparing action of diets with greater energy content. Still, as compared to starving conditions, such therapy significantly reduces the wasting of vital tissue substrates and does not prompt a dangerous increase in cardio-respiratory demands.

## Figures and Tables

**Table 1:** Determinants of energy consumption for every mole of substrate.

Substrate	O_2_ Consumed (L)	CO_2_ Produced (L)	RQ	Energy Yield (kcal)	ATP Yield (mol)
Glucose	134	134	1	670	36
Palmitate	515	358	0.7	2400	132
Amino acids	114	92	0.8	479	23

The table shows the oxygen consumption and carbon dioxide production derived from the complete oxidation of one mole of carbohydrates, lipids, and amino acids, as well as the respiratory quotient (RQ, i.e., the ratio of carbon dioxide produced and oxygen consumed) and the energy yield (in kcal and moles of ATP produced).

**Table 2:** Summary of studies related to caloric and protein load and clinical outcomes.

Study	Study Design	Comparator	Main Finding
Alberda 1999 [34]	Observational cohort study; 2772 mechanically ventilated patients	Calories prescribed	Provision of higher calories was associated with reduced 60-day mortality (OR 0.76 for every 1000 kcal/day provided) Sub-analysis for BMI classes suggested significant effects for patients with a BMI <25 or ≥35 kg/m^2^
Villet 2005 [31]	Prospective observational study; 48 patients	Energy balance	The cumulative negative energy balance was significantly correlated with the length of ICU stay and the number of infectious complications
Dvir 2006 [35]	Prospective observational study; 50 patients	Energy balance	The cumulative negative energy balance during ICU stays was significantly correlated with a higher rate of occurrence of ARDS, renal failure requiring surgery, and the total complication rate
Heyland 2011 [36]	Prospective, multi-institutional audit; 7872 mechanically ventilated patients	Calories prescribed	Patients who received >2/3 of their caloric prescription had lower mortality than those receiving <1/3 of their prescription (OR 0.67)
Weijs 2012 [37]	Prospective observational cohort study; 886 patients	Provision of both the protein and energy target	Provision of a protein target (defined as 1.2 g/kg) was associated with a reduced 28-day mortality (The hazard ratios for the energy target and protein + energy target were 0.83 (0.67–1.01) and 0.47 (0.31–0.73))
Allingstrup 2012 [38]	Prospective observational cohort study; 113 patients	Proteins prescribed	A significantly decreased hazard ratio of ICU mortality was associated with increased protein provision (HR 0.98 for every g/day of protein prescribed)
Wei 2015 [29]	Retrospective analysis of prospectively collected data from a multicenter RCT; 475 patients	Proportion of received/prescribed calories during the first 8 days	Greater amounts of nutritional intake were associated with longer survival times and faster physical recovery to 3 months
Zusman 2016 [39]	Retrospective; 1171 patients	Outcome vs. the percentage of administered calories	The % AdCal/REE had a significant non-linear association with mortality after adjusting for other variables. Increasing the percentage from 0 to 70% resulted in a hazard ratio of 0.98, pointing to reduced mortality, while increases above 70% suggested an increase in mortality
Compher 2017 [40]	Prospective; 202 patients	Nutritional risk and nutritional intake	In high-risk but not low-risk patients, mortality was lower with greater protein and energy intake
Koekkoek 2019 [41]	Retrospective; 455 patients	Low vs. high protein intake	Time-dependent association of protein intake and mortality; low protein intake (<0.8 g/kg/day) before day 3 and high protein intake (>0.8 g/kg/day) after day 3 were associated with lower 6-month mortality compared to patients with overall high protein intake
Hartl 2022 [42]	Retrospective; 16,489 patients	Protein intake	In comparison with an exclusively low-protein diet, a late standard protein diet was associated with a lower hazard of in-hospital death: minimum 0.75 (95% CI 0.64, 0.87), and a higher hazard of live hospital discharge: maximum HR 1.98 (95% CI 1.72, 2.28)

BMI = body mass index; ICU = Intensive Care Unit; HR = High Rate; RCT = randomized control trial; REE = rest energy expenditure; CI = confidence interval.

## Data Availability

The data presented in this study are available upon request from the corresponding author. The data are not publicly available due to privacy and ethical restrictions.
